# Testing a mediation model of teacher caring, grit, and student wellbeing in English as a foreign language students

**DOI:** 10.3389/fpsyg.2023.1260827

**Published:** 2023-09-06

**Authors:** Guanbing Zhou

**Affiliations:** College of Education, Zhongyuan Institute of Science and Technology, Zhengzhou, China

**Keywords:** perceived teacher caring, student wellbeing, Chinese EFL students, grit, mediating role, teacher-student relationships

## Abstract

**Introduction:**

This study delves into the influence of perceived teacher caring on the wellbeing of Chinese English as a Foreign Language (EFL) students, with a specific focus on the potential mediating effect of grit. With a sample size of 748 EFL students hailing from diverse Chinese universities, we aimed to shed light on the connections between perceived teacher caring, student wellbeing, and the mediating factor of grit.

**Methods:**

To address our research questions, we collected data through self-report questionnaires that gauged perceived teacher caring, student wellbeing, and levels of grit. By utilizing structural equation modeling, we were able to rigorously assess both the direct and indirect impacts of teacher caring on student wellbeing.

**Results:**

Our analysis uncovered a noteworthy and positive correlation between perceived teacher caring and student wellbeing. Furthermore, our findings indicated that grit plays a significant mediating role in this relationship. This suggests that students who exhibit higher levels of grit are more inclined to experience heightened levels of wellbeing.

**Discussion:**

The outcomes of this study underscore the significance of nurturing positive teacher-student relationships within the EFL context. The identification of grit as a mediator emphasizes its role in fostering enhanced student wellbeing. These findings collectively emphasize the intertwined nature of teacher caring, grit, and student wellbeing. Educators should consider these insights for their teaching practices, while researchers can use this study as a foundation for further investigations in this domain.

## Introduction

The incorporation of positive psychology into second language acquisition (SLA) research has shed light on the significant benefits that positive emotions and individual differences can bring to both language learners and instructors (Dewaele et al., 2019; Li, 2020; Fathi et al., 2023a). Within language classrooms, the quality and quantity of teacher-student interactions play a crucial role, and the emotional and relational dynamics between second language (L2) instructors and students underscore the importance of employing positive interpersonal indicators to influence L2 students positively and foster the development of favorable academic attitudes, such as engagement (MacIntyre et al., 2016; Mercer and Gkonou, 2020; Xie and Derakhshan, 2021; Fathi et al., 2023b).

Furthermore, the concept of well-being, as another key aspect of positive psychology, has been linked to personal growth and enhanced health outcomes (Anderson and Graham, 2016; Wang et al., 2021). Well-being encompasses feelings of joy, fulfillment, and good health, reflecting a strong sense of purpose or meaning, sound mental well-being, effective stress management, and high levels of life satisfaction (Bücker et al., 2018; Braun et al., 2020; Collie, 2022; Nalipay et al., 2022). Despite the growing recognition of the positive influence of teacher-student interactions and well-being in language learning environments (Longobardi et al., 2021; Fabris et al., 2022), there remains a gap in understanding the specific relationship between perceived teacher caring and student well-being in the context of Chinese English as a Foreign Language (EFL) students.

Teacher care, a crucial variable in this study, plays a pivotal role in the context of L2 education, where instructors profoundly influence students’ perceptions of equality and engagement (Gkonou and Miller, 2019; Pishghadam et al., 2019; Lavy and Naama-Ghanayim, 2020; Yan, 2021). The concept of care, originally introduced by Noddings (1984), revolves around the relationship between the caregiver and the one receiving care. In the context of education, teacher care refers to teachers’ actions that foster positive relationships with their students and demonstrate awareness and sensitivity to their emotional needs (Lewis et al., 2012; Gabryś-Barker, 2016; Derakhshan et al., 2022). In other words, teacher care encompasses instructor-initiated activities that nurture close interpersonal connections with students, reflecting perceptions of closeness, responsiveness to students’ needs, and compassion between teachers and pupils (Noddings, 2012; Miller and Gkonou, 2023). The significance of teacher care is underscored by Laletas and Reupert (2016) who assert that “neither pedagogy nor discipline strategy would be effective without care” (p. 496). Considering its undeniable importance, exploring the impact of teacher care on student wellbeing in the context of Chinese EFL students becomes a critical avenue for research, and this study aims to fill this gap.

The third variable examined in this study is grit, which encompasses qualities of perseverance, tenacity, and enthusiasm essential for accomplishing long-term goals. Grit can be viewed as a multifaceted construct, comprising both persistence in effort and constancy in interest (Duckworth et al., 2007; Teimouri et al., 2022). Persistence of effort involves a willingness to make continuous efforts and work diligently when faced with challenges and setbacks, while consistency of interest relates to maintaining enthusiasm and interest in pursuing goals, even in the face of difficulties (Duckworth and Quinn, 2009; Li, 2020; Sudina et al., 2021). Notably, grit is malleable, meaning that it can be strengthened through guidance and interventions in educational settings. This adaptability of grit holds particular promise in language classrooms, where L2 instructors can support students in developing their grit to navigate challenges during the language learning process (Clark and Malecki, 2019; Sudina and Plonsky, 2021).

Although previous studies have separately investigated the variables mentioned above, this study aims to explore the impact of perceived teacher caring on student wellbeing in EFL students while considering the role of grit. In other words, there’s still a lack of understanding regarding the specific relationship between perceived teacher caring and student wellbeing in the context of EFL students, especially in China. This study aims to bridge this gap by focusing on the unique challenges and dynamics present in the EFL learning environment in China. Another aspect that has received limited attention in the education context is the role of grit as a mediator between perceived teacher caring and student wellbeing. This research seeks to shed light on how grit might influence the link between teacher-student relationships and students’ psychological and emotional wellbeing in EFL settings. Via addressing these gaps, the study offers valuable insights for educators and policymakers, helping them create more supportive and enriching learning experiences for EFL students, ultimately fostering their overall wellbeing and academic success.

## Literature review

### Positive psychology

According to the flourishing of positive psychology in SLA, researchers have adopted a constructive approach to enhance the physical, personal, and interpersonal aspects of life for L2 instructors and learners (MacIntyre et al., 2016; Fathi et al., 2023b). This positive orientation is believed to positively influence students’ interest in achieving academic goals in the L2 setting (Dewaele et al., 2019; Zhang et al., 2023). Li (2020) highlights a “positive renaissance” in SLA studies over the past decade, prompting a shift from focusing on negative emotions (e.g., boredom and anxiety) to positive emotions (e.g., engagement and grit) (Resnik and Dewaele, 2020; Cheng and Liu, 2022; Mystkowska-Wiertelak, 2022; Teimouri et al., 2022; Zhang et al., 2022; Fathi et al., 2023a). In alignment with this trend, Mercer et al. (2018) introduced the concept of “Positive Language Education,” which combines language learning with positive pedagogy, advocating for kindness and compassion to foster wellbeing in the L2 environment (Mohammad Hosseini et al., 2022). This psychological support enhances feelings of closeness and involvement for both L2 instructors and learners (Mercer and Dörnyei, 2020).

Xie and Derakhshan (2021) propose that instructors can enhance students’ positive educational outcomes by cultivating positive teacher-student interpersonal factors. They highlight the significance of establishing a pleasurable educational setting by demonstrating regard and concern for students, promoting positive teacher-student connections, and cultivating enthusiasm for the language being taught. Given the emotionally charged nature of language instruction, positive emotions serve as favorable indicators of students’ language acquisition. Language teachers play a pivotal role in boosting students’ sense of fulfillment, broadening their perspectives, and fostering language learning (Dewaele et al., 2019; Derakhshan et al., 2022; Mystkowska-Wiertelak, 2022).

### Well-being

Student well-being in educational contexts is of paramount importance as it directly influences students’ overall development, academic success, and lifelong learning outcomes (Upsher et al., 2022). A positive and supportive learning environment plays a crucial role in fostering students’ emotional, psychological, and social well-being (Bücker et al., 2018). When students feel cared for, respected, and valued by their teachers and peers, they are more likely to be engaged, motivated, and eager to participate in classroom activities (Anderson and Graham, 2016; Morinaj and Hascher, 2019). This, in turn, enhances their academic performance and helps create a conducive atmosphere for effective learning. Moreover, student well-being is closely linked to their mental health and resilience, enabling them to cope with stress, challenges, and setbacks, and promoting a sense of belonging and connectedness within the educational community (Harding et al., 2019; Braun et al., 2020).

Collie (2022) identifies happiness, propensity, and health as key elements contributing to well-being. Ryff (1989) outlines six crucial components, including accepting oneself, having a purpose in life, personal development, positive relationships with others, independence, and environmental mastery, that collectively define well-being. Seligman’s (2011) well-being model, PERMA (Positive Emotion, Engagement, Relationships, Meaning, and Accomplishment), emphasizes the most significant aspects of well-being (Mercer and Gregersen, 2020). Talbot and Mercer (2018) emphasize that teachers with high levels of well-being are more likely to be successful in their teaching practice, exhibit greater engagement, and effectively navigate challenges in their profession.

In the context of language acquisition and instruction, understanding and fostering the health of both students and instructors in L2 education is vital, as highlighted by Wang et al. (2021). Well-being has been linked to various positive outcomes, such as increased enjoyment of foreign languages among students, improved emotion regulation, and enhanced fulfillment in the classroom for instructors (Greenier et al., 2021). Dewaele et al. (2018) conducted research on EFL/ESL instructors, exploring the connections between emotional intelligence, teaching experience, knowledge, and instructional methods, while considering well-being as a reliable psychological construct. Their findings suggest that enhancing the emotional intelligence of EFL instructors can positively impact their passion for the language, pedagogical expertise, and overall professional well-being. Additionally, the study revealed a positive correlation between instructors’ engagement with learners and their well-being. MacIntyre et al. (2019) further investigated language instructors’ well-being using a mixed-methods approach, aligning with Seligman’s PERMA model and Goldberg’s Big Five Personality model. The study concluded that perceived positive emotions, engagement, positive relationships, meaning, and accomplishment significantly influenced language instructors’ well-being. Furthermore, language instructors’ well-being was found to be positively associated with agreeableness, conscientiousness, emotional stability, intellect, and extraversion. However, qualitative analysis highlighted a severe workload and limited financial support as primary factors endangering language instructors’ well-being.

### Teacher care

Teacher care plays a pivotal role in the educational journey, shaping students’ academic and personal growth by fostering a positive and nurturing atmosphere in the classroom (Lewis et al., 2012; Ramberg et al., 2019). This aspect encompasses emotional support, attention, and empathy extended by teachers to their students (Teven, 2007; Longobardi et al., 2021). Creating a learning environment grounded in teacher care involves acknowledging individual students’ unique needs, building trust, and cultivating rapport within the learning community (Teven and McCroskey, 1997). The significance of teacher care is underscored by a growing body of research highlighting its positive effects on various student outcomes, including heightened engagement, motivation, and academic achievement (Muller, 2001; Sun, 2021). When students sense genuine care and value from their teachers, a sense of security and support emerges, contributing to an enriched learning experience (Lavy and Naama-Ghanayim, 2020). Furthermore, teacher care is intricately connected to students’ emotional and social development, fostering a sense of belonging and connection within the educational setting (Miller and Gkonou, 2023).

Recent investigations have accentuated the instrumental role of teacher-student relationships and a positive classroom atmosphere in enhancing students’ outcomes. For instance, Sulla and Rollo (2023) conducted a study exploring the effect of a short course on Italian primary school teachers’ praise rates and its impact on pupils’ on-task behavior. Their findings demonstrated the direct correlation between teachers’ nurturing approach and students’ improved behavior, reaffirming the role of teacher care in cultivating a conducive learning environment.

In the context of language education, teacher care holds particular importance. Mercer and Gkonou (2020) emphasize the significance of teacher care in fostering effective teacher-learner connections within English language classrooms. Moskowitz’s (1978) study illustrates how caring actions by language instructors can catalyze L2 improvement, emotional growth, and well-being among students within the framework of positive psychology. The concept of “teacher clarity” further aligns with teacher care, promoting enhanced communication between instructors and students, which leads to improved comprehension and engagement, thereby fostering a strong teacher-student connection (Yan, 2021). Additionally, researchers like Cakir (2015) and Xie and Derakhshan (2021) have defined teacher immediacy as a collection of verbal and nonverbal strategies employed by instructors to establish a sense of connection with their students. Also, the recent investigation by Sulla and Rollo (2023) further attests to the direct influence of a nurturing teacher-student relationship on students’ behavior and engagement. As we delve into the intricacies of teacher-student relationships and classroom dynamics, it becomes evident that teacher care serves as a cornerstone for fostering a positive and productive learning environment.

According to Pishghadam et al. (2015), L2 teacher care comprises three key elements: unbiased relationships with students, appropriate feedback, and teacher stroke. Biased relationships occur when language instructors favor some students over others, leading to unequal treatment (Derakhshan et al., 2022). Language instructors demonstrate care through active listening and providing feedback to students, offering information on their performance in language acquisition tasks (Noddings, 2012). The final aspect of teacher care is teacher stroke, which involves language instructors taking actions to demonstrate their awareness of students’ presence and concern for them in the L2 setting (Pishghadam et al., 2015). In a study by Pishghadam et al. (2019), the impact of teacher stroke on undergraduate TEFL college students’ willingness to participate in L2 classes showed a statistically significant positive association between the two elements. Additionally, Banse and Palacios (2018) found that when students perceived high levels of care and control from instructors, grit was significantly connected to their success in English and language arts.

### Grit

Grit is a non-cognitive personality trait associated with academic and personal achievements, encompassing two components: perseverance of efforts (PE) and consistency of interest (CI) (Duckworth et al., 2007; McCain, 2017; Credé, 2018). PE involves the ability to persist and achieve competence despite facing setbacks, while CI is essential for sustained practice and mastery, both of which significantly impact achievement (Credé et al., 2017; Duckworth et al., 2021). Within the context of L2 education, grit has been linked to accomplishment, enjoyment, and willingness to communicate (Akos and Kretchmar, 2017; Khajavy et al., 2021; Alamer, 2022; Teimouri et al., 2022; Mikami, 2023).

However, it is important to note that there has been a measure of controversy surrounding the measurement of grit. Notably, while the two-factor structure (PE and CI) has been validated in numerous studies (Duckworth et al., 2007; McCain, 2017; Credé, 2018), some recent research has proposed a one-dimensional structure of grit (Sulla et al., 2018; Postigo et al., 2021). For instance, Postigo et al. (2021) and Sulla et al. (2018) have reported findings consistent with a single-dimensional interpretation of grit. This controversy has also been recognized by the authors themselves (Duckworth et al., 2021) in a commentary on the factor structure of grit. This variability in conceptualization and measurement could potentially introduce a degree of ambiguity in certain aspects of grit’s nature and its impact on various outcomes. Therefore, it is prudent to acknowledge that while the two-factor structure of grit is widely accepted, ongoing debates about its dimensionality underscore the complexity and evolving nature of this construct.

Recent investigations have illuminated the role of grit in academic contexts, reinforcing its significance as a predictor of achievement. For instance, Sulla et al. (2022) conducted a study exploring university students’ online learning. Their findings underscored the positive influence of grit on academic performance, suggesting that students with higher levels of grit exhibited greater resilience and adaptability in the face of challenging circumstances. Furthermore, Yang et al. (2022) delved into the contribution of buoyancy and self-efficacy to L2 grit among EFL learners. Their results indicated that these factors, alongside grit, collectively contribute to learners’ persistence and dedication, offering valuable insights into the multifaceted nature of grit’s impact on academic endeavors.

In an innovative investigation, Lake (2013) explored the attributes of perseverance using the general domain scale in the L2 context. The outcomes revealed that learners with higher levels of determination reveal a greater willingness to dedicate time and effort to mastering L2. Similarly, Changlek and Palanukulwong (2015) delved into the impact of grit and motivation on the English language proficiency of 180 Thai students. They discovered a robust and positive association between grit and motivation, particularly among the most accomplished individuals. Kramer et al. (2018) found a moderate correlation between participants’ language proficiency and grit in Japanese learners. Moreover, Robins (2019) revealed a significant connection between grit and the EFL students’ GPA.

Wei et al. (2019) investigated the relationship between grit and English language achievement among Chinese secondary school students, revealing a weak but positive correlation between the two. Similarly, Wei et al. (2020) explored socio-biographical factors, such as multilingualism, age, and gender, and their connection to L2 grit in the Chinese context. Elahi Shirvan et al. (2021) conducted a longitudinal study on L2 grit and foreign language enjoyment, finding a growing pattern of association between these two components. Li and Dewaele (2021) emphasized the significance of the learning environment and observed that overall grit in the context of online English language training affects classroom anxiety in foreign language learning. Additionally, Resnik et al. (2021) discovered that L2 grit served as a correlate of pleasure in learning a foreign language.

Regarding domain-specific PE and CI, Sudina and Plonsky (2021) found that PE demonstrated better criterion validity for students’ accomplishment in the language domain, compared to CI, in their examination of the relationship between language learning grit and L2 and L3 achievement. Khajavy and Aghaee (2022) tested the relationship between L2 grit and L2 achievement, considering other related factors, and found that while PE alone could predict L2 achievement, neither PE nor CI could do so when emotions and personal bests were taken into account. Credé and Tynan (2021) assert that persistence and passion are essential for language acquisition, but they are not sufficient on their own, suggesting the need for considering additional factors in the study of L2 grit (Alamer, 2021, 2022; Oxford and Khajavy, 2021).

## The structural model

**Figure 1:**
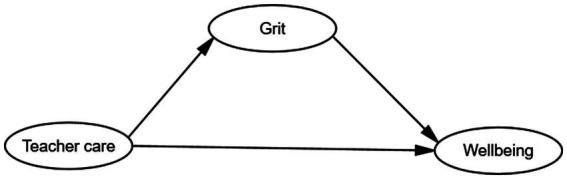
The structural model.

Figure 1 presents the hypothesized model for the study, which explores the relationship between perceived teacher caring, student wellbeing, and the mediating role of grit among Chinese EFL students. The model posits that perceived teacher caring directly impacts student wellbeing and also indirectly influences wellbeing through the mediating variable of grit.

It is hypothesized that higher levels of perceived teacher caring will be positively associated with greater student wellbeing, based on previous research emphasizing the significance of teacher-student relationships in enhancing student outcomes (Lewis et al., 2012; Ramberg et al., 2019; Lavy and Naama-Ghanayim, 2020; Longobardi et al., 2021; Lin et al., 2022). Grit, the proposed mediating variable, is expected to play a crucial role in the relationship between perceived teacher caring and student wellbeing. Grit encompasses the traits of perseverance and passion for long-term goals, which may enable students to better cope with challenges and maintain a sense of purpose and determination in their academic pursuits (Duckworth et al., 2007; Credé and Tynan, 2021; Sudina et al., 2021). The model posits that perceived teacher caring might positively influence students’ grit levels, as caring teachers are likely to foster a supportive and encouraging learning environment that nurtures students’ resilience and perseverance (Muller, 2001; Lewis et al., 2012).

Furthermore, it is hypothesized that higher levels of grit will, in turn, positively impact student wellbeing. Gritty individuals are believed to exhibit greater psychological resilience, higher levels of engagement, and a more positive outlook on life, which can contribute to overall wellbeing (Credé et al., 2017; Duckworth et al., 2021). The hypothesized relationships between perceived teacher caring, grit, and student wellbeing are supported by previous research emphasizing the role of teacher caring in fostering positive student outcomes and the positive impact of grit on academic achievement and emotional well-being (Teven and McCroskey, 1997; Teven, 2007; Pianta et al., 2012; Sun, 2021).

## Methods

### Participants and procedure

A carefully planned stratified sampling technique was employed to ensure the representation of a diverse group of participants. The initial survey encompassed 748 college students drawn from six universities across distinct regions of China: northeastern, eastern, and northwestern. The inclusion of one key national university and one ordinary university from each region contributed to a well-balanced selection, enhancing the robustness of our sample.

The sample consisted of freshmen and sophomores who were pursuing majors other than English. Out of the total sample, 426 participants (approximately 57%) identified themselves as female, while 322 individuals (approximately 43%) identified as male. Participants’ ages ranged from 19 to 21 years, resulting in a homogeneous age group for analytical purposes.

In China, college-level English courses are typically included as part of the curriculum for all students, regardless of their majors. These English courses serve a twofold purpose: first, to cultivate and enhance students’ English language proficiency, and second, to facilitate effective cross-disciplinary communication skills. To ensure the sample’s homogeneity, juniors and seniors were not included as they were not obligated to take college English as a mandatory course. Additionally, English majors were excluded to mitigate potential confounding variables stemming from variations in course selection and learning outcomes. This approach further bolstered the representativeness of the sample, aligning with the study’s objectives.

The participants’ involvement in the study was purely voluntary and had no bearing on their academic performance. The recruitment process adhered to ethical guidelines and was conducted with transparency. The data collection period spanned for about 5 weeks, during which access to the participating universities was granted through established academic connections. The data collection process was integrated into participants’ regular classes, ensuring minimal disruption to their academic schedules. Questionnaires, organized as booklets, were distributed to participants. Before commencing the questionnaire, researchers provided a comprehensive overview of the study’s aims, emphasizing confidentiality and the voluntary nature of participation. Informed consent was obtained from all participants prior to questionnaire administration. Clear instructions were reiterated verbally to participants, emphasizing the importance of basing responses on recent and/or memorable experiences from English classes. Approximately 15 min were allocated for questionnaire completion, ensuring a manageable timeframe for participants.

### Instruments

#### Student well-being

Student well-being was measured using the Satisfaction with Life Scale (SWL; Diener et al., 1985). The SWL consists of five items assessing individuals’ satisfaction with their life, such as “I am satisfied with my life.” Participants rated their agreement with each item on a seven-point Likert-type scale, ranging from 1 (strongly disagree) to 7 (strongly agree). The internal consistency of the scale was deemed satisfactory, with a Cronbach’s alpha coefficient of 0.81.

#### Teacher care

The Teacher Care Scale (TC-S) developed by Gallagher et al. (2019) was utilized to assess the positive aspects of teacher-student relationships, particularly teacher caring. The TC-S consists of **six** items that measure students’ perceptions of their teachers’ support and level of care. Participants responded to these items using a 5-point Likert scale ranging from 1 (not true at all) to 5 (very true). To calculate a composite score for perceived teacher caring, the mean of the six items was calculated for each student. Gallagher et al. (2019) reported a high level of internal consistency for this scale (*α* = 0.88), indicating its reliability.

#### Grit

The L2 grit scale developed by Teimouri et al. (2022) was employed to measure foreign language grit. This scale consists of 9 items that assess two sub-scales: consistency of interest (CI) and perseverance of effort (PE). Participants rated their agreement with each item on a five-point Likert scale. The CI sub-scale includes 4 items that capture the fluctuation of interest in learning English over time. The PE sub-scale comprises 5 items that reflect the individual’s level of dedication and effort in mastering English pronunciation. Teimouri et al. (2022) reported satisfactory construct validity and demonstrated acceptable reliability coefficients for the different sub-scales of this scale. The reliability of the scale in this study was satisfactory (*α* = 0.84).

#### Instrument pre-testing

To ensure a comprehensive understanding of the instrument items, a meticulous pre-testing or initial piloting phase was conducted prior to the primary data collection. This crucial step was undertaken to align the research instruments with the linguistic capacities of our participants.

During this pre-testing phase, a group of 35 participants thoughtfully engaged with the questionnaires and provided valuable feedback. This iterative process allowed for a thorough examination of the clarity of each item and facilitated the identification of potential areas of confusion. The feedback gathered during the pre-testing phase affirmed the instruments’ clarity and intelligibility for the participants. The positive responses received from participants underscored their unequivocal understanding of the questionnaire items and their ability to provide insightful and meaningful responses.

### Data collection

The data collection process for this study involved the distribution of questionnaires to participants during their regular classes. The questionnaires were provided in a physical format, organized as a booklet to facilitate easy completion. Prior to distributing the questionnaires, the researchers provided a thorough explanation of the research objectives and reassured the participants that their participation was voluntary and confidential. It was explicitly emphasized that their involvement in the study would have no impact on their academic performance. Only those who provided informed consent were given the questionnaire to complete. To ensure clarity and understanding, the researchers verbally reiterated the instructions written on the questionnaire. Participants were instructed to base their responses on their experiences in a recent and/or memorable English class. They were allocated approximately 15 min to complete the questionnaire.

### Data analysis

Data analysis for this study was conducted using Maximum Likelihood Estimation (MLE) in SPSS (Version 26) and Amos (Version 26). Before conducting the analysis, a comprehensive assessment of the data was performed to address missing data, outliers, and multivariate normality. The rate of missing data was minimal, approximately 0.08%, and the Missing Completely at Random (MCAR) test using Little’s test confirmed the complete randomness of the missing data (χ^2^ = 562.78, *p* = 0.387). To handle the missing data, Expectation Maximization (EM), a robust and suitable estimation technique for Structural Equation Modeling (SEM), was utilized for data imputation (Little and Rhemtulla, 2013).

Univariate outliers were identified by examining scatter plots and Z-standardized values. The assumption of normality was met, as indicated by skewness and kurtosis values within the acceptable range of −2 and + 2 (Kline, 2023) (see Table 1). Furthermore, multivariate outliers were assessed using Mahalanobis distances (Tabachnick et al., 2013). Following these initial checks, a total of 11 cases were excluded from the analysis, resulting in a final dataset comprising 737 participants. To ensure the construct validity of the questionnaires across diverse contexts and specialized fields, three separate Confirmatory Factor Analyses (CFAs) were conducted. The results indicated satisfactory model fit, supporting the validity of the questionnaires (Table 2). Finally, SEM was employed to analyze the mediating role of teacher grit in the relationship between teacher growth mindset and teacher well-being. Several fit indices were used to assess the model’s goodness of fit. The χ^2^-goodness of fit to the degree of freedom (df) ratio was evaluated, with a good fit considered when the ratio was less than 3, and the value of *p* greater than 0.05. Additionally, the Goodness of Fit Index (GFI) and Comparative Fit Index (CFI) values were examined, with values of 0.90 or higher indicating a good fit. Furthermore, the Root-Mean-Square Error of Approximation (RMSEA) was assessed, with a value of less than 0.08 considered indicative of a good fit, as well as the Standardized Root-Mean-Square Residual (SRMR), with a value of less than 0.10 (Hu and Bentler, 1999; Tabachnick et al., 2013; Kline, 2023).

**Table 1:** Descriptive statistics.

Constructs	1	2	3
1. Teacher caring	1		
2. Grit	0.53**	1	
3. Wellbeing	0.41**	0.46**	1
4. Mean	3.66	3.26	4.03
5. SD	0.71	0.79	0.94
6. Skewedness	−0.46	−0.14	−0.16
7. Kurtosis	−0.52	−0.56	−0.47

*p < 0.05, **p < 0.01.

**Table 2:** Results of CFA.

	CMIN	DF	CMIN/DF	*P*	CFI	RMSEA	SRMR	α
Wellbeing	85.124	48	1.774	<0.001	0.956	0.073	0.043	0.81
Care	110.352	72	1.531	<0.001	0.972	0.041	0.035	0.88
Grit	160.745	94	1.709	<0.001	0.967	0.049	0.039	0.84

## Results

In the present study, descriptive statistics were computed to examine the means and standard deviations of the three variables. The mean scores for teacher caring were 3.82 ± 0.73 for males and 3.91 ± 0.67 for females. Regarding L2 grit, the mean scores were 3.19 ± 0.78 for males and 3.22 ± 0.72 for females. For the variable of wellbeing, the mean scores were 4.18 ± 0.88 for males and 4.16 ± 0.85 for females. Independent samples t-tests were conducted to compare the means between genders. The results indicated no significant gender differences in wellbeing [*t*(746) = −0.764, *p* = 0.389], teacher caring [*t*(746) = −0.315, *p* = 0.679], and L2 grit [*t*(746) = −0.622, *p* = 0.602].

Table 2 provides an overview of the first-order confirmatory factor analyses (CFAs) conducted to assess the reliability and goodness of fit of the wellbeing, care, and grit measurement scales. The results of the CFAs demonstrate that all three measurement scales exhibit favorable model fit. Additionally, the reliability coefficients (α) of the wellbeing, care, and grit scales are reported as 0.81, 0.88, and 0.84, respectively, indicating satisfactory internal consistency. Overall, the results of the CFAs and the reliability indices suggest that the measurement scales possess favorable psychometric properties, supporting their suitability for measuring the corresponding constructs.

Table 1 illustrates the descriptive statistics for the key constructs of teacher caring, grit, and wellbeing in the study. The interrelationships among these constructs were examined, shedding light on their associations and providing important insights into the research variables. The construct of teacher caring exhibited significant positive correlations with both grit [*r*(746) = 0.53, *p* < 0.01] and wellbeing [*r*(746) = 0.41, *p* < 0.05], as hypothesized. Similarly, grit showed a significant positive correlation with wellbeing [*r*(746) = 0.46, *p* < 0.01]. These correlation coefficients indicate the strength and direction of the relationships between the constructs, supporting the underlying theoretical framework. In terms of the descriptive statistics, the mean score for teacher caring was 3.66 (SD = 0.71), while the mean scores for grit and wellbeing were 3.26 (SD = 0.79) and 4.03 (SD = 0.94), respectively.

Afterwards, SEM was used to examine the mediating role of L2 grit in the association between teacher caring and student wellbeing. To evaluate the indirect effects, a resampling technique known as bootstrap resampling was utilized with 500 iterations. This widely accepted method in SEM allows for the assessment of the sampling distribution and facilitates the examination of the indirect effects (Hayes, 2009). The model’s fit to the data, investigating the mediating role of grit in the relationship between perceived teacher care and wellbeing, was assessed using several fit indices. The analysis revealed that the proposed model demonstrated a good fit to the data based on the following fit indices: χ^2^/df = 1.613, CFI = 0.949, TLI = 0.944, IFI = 0.947, RMSEA = 0.039, and SRMR = 0.043. These indices indicate that the model adequately captures the relationships among the variables and is consistent with the observed data. The goodness-of-fit indices suggest that the proposed model provides an accurate representation of the theoretical framework being examined.

**Figure 2:**
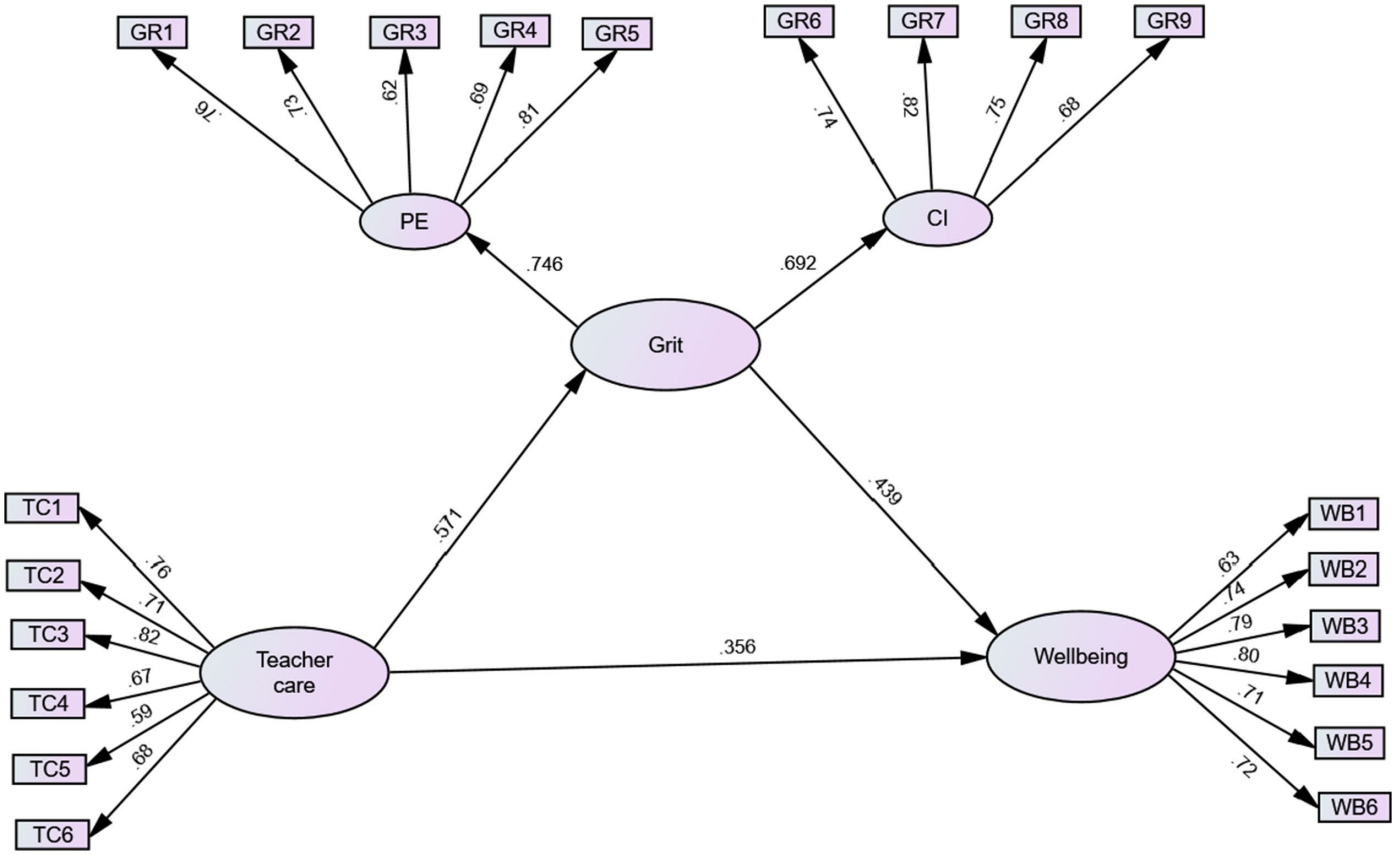
The final model.

To better understand the relationships within the proposed model, Figure 2 presents the standardized parameter estimates. These estimates provide information about the strength and direction of the paths connecting teacher caring, L2 grit, and student wellbeing.

Also, Table 3 presents the path estimates of the structural model examining the effect of perceived teacher caring on student wellbeing in EFL students, with grit as a potential mediator. In terms of the direct effects, the path from teacher caring to student wellbeing yielded a significant positive effect (*β* = 0.356, *p* < 0.001). Similarly, the path from teacher caring to grit showed a significant positive effect (*β* = 0.571, *p* < 0.001). Moreover, the path from grit to student wellbeing was also found to have a significant positive effect (*β* = 0.439, *p* < 0.001).

**Table 3:** Path estimates of the structural model.

Model pathways	B	SE	*β*	*P*	95% CI	
					Lower bound	Upper bound
Direct effects						
Teacher caring→ wellbeing	0.426	0.168	0.356	<0.001	0.123	0.729
Teacher caring→ grit	0.721	0.163	0.571	<0.001	0.403	0.923
Grit→ wellbeing	0.539	0.072	0.439	<0.001	0.402	0.667
Indirect effect						
Teacher caring → grit → wellbeing	0.343	0.091	0.250	<0.001	0.153	0.486

Regarding the indirect effect, the pathway from teacher caring to grit to student wellbeing revealed a significant positive effect, indicating that grit partially mediates the relationship between teacher caring and student wellbeing (*β* = 0.250, *p* < 0.001). The combined influence of teacher care and L2 grit, calculated by summing the squared path coefficients from teacher care to student wellbeing and from grit to student wellbeing ( $0.356^{2}+0.439^{2}=0.319$ 7), accounted for 31.97% of the variability observed in student wellbeing, suggesting that there are additional factors beyond teacher care and grit that contribute to the remaining variance. These findings suggest that perceived teacher caring has a direct positive influence on student wellbeing and also indirectly affects student wellbeing through the mediating role of grit. The statistically significant coefficients and their corresponding confidence intervals provide support for the proposed structural model.

Moreover, an additional analysis was conducted to assess the structural invariance of the mediation model and determine whether the structural path coefficients differed significantly between male and female participants (see Table 4). The findings revealed that the recommended mediation model demonstrated a good fit for both gender groups. A multi-group invariance analysis conducted across gender indicated that both the restricted model (χ^2^/df = 1.480, CFI = 0.936, TLI = 0.935, RMSEA = 0.042, SRMR = 0.072) and the unrestricted model (χ^2^/df = 1.478, CFI = 0.937, TLI = 0.929, RMSEA = 0.040, SRMR = 0.071) exhibited good fit with the data. The χ^2^ difference test between the two models (Δχ^2^ = 4.442, Δdf = 3, *p* = 0.216) suggested that the model coefficients in the recommended mediation model were invariant across gender.

**Table 4:** Structural invariance analysis and gender-specific SEM results.

Model	χ2/df	*p*	CFI	TLI	RMSEA	SRMR
Restricted model	1.480	< 0.001	0.936	0.935	0.042	0.072
Unrestricted model	1.478	< 0.001	0.937	0.929	0.040	0.071
Male	1.406	< 0.001	0.939	0.938	0.050	0.068
Female	1.453	< 0.001	0.934	0.932	0.047	0.064

Furthermore, separate structural equation modeling analyses were conducted for male and female learners, which indicated acceptable fit for both groups. Specifically, for male learners, the model demonstrated good fit (χ^2^/df = 1.406, *p* < 0.001, CFI = 0.939, TLI = 0.938, RMSEA = 0.050, SRMR = 0.068), while for female teachers, the model also showed good fit (χ^2^/df = 1.453, *p* < 0.001, CFI = 0.934, TLI = 0.932, RMSEA = 0.047, SRMR = 0.064). These results suggest that there were no significant gender differences observed in the direct and indirect effects of teacher caring on wellbeing. Moreover, the mediating role of grit was found to be strong for both genders.

## Discussion

The present study aimed to investigate the impact of perceived teacher caring on student wellbeing in Chinese EFL students, with a particular focus on the mediating role of grit. The findings provide valuable insights into the relationship between teacher-student dynamics and student wellbeing, shedding light on the role of individual characteristics such as grit in this process.

The relationship between teacher care and students’ well-being was investigated, revealing that students’ perception of teacher caring directly influences their well-being. Consistent with Mercer and Gkonou (2020) and Noddings (2012), students reported experiencing feelings of intimacy, compassion, and a sense of mutual trust and connection with their teachers. These positive teacher-student relationships fostered a sense of commitment and engagement in foreign language learning, aligning with the principles of positive psychology in L2 education (Pianta et al., 2012; Dewaele et al., 2019; Wang et al., 2021; Zhang et al., 2022). Creating a pleasant L2 classroom environment through compassionate and kind teacher behaviors encourages student engagement and participation (Mohammad Hosseini et al., 2022). This finding is also justified in light of social support theory which suggests that when students perceive their teachers as caring and supportive, they have access to a valuable source of emotional and instrumental support. This support buffers the negative impact of stressors, promotes adaptive coping strategies, and ultimately contributes to increased wellbeing (Cohen and Wills, 1985). When students perceive their teachers as caring and supportive, they feel valued, respected, and understood, leading to positive emotional experiences and a greater sense of wellbeing. These positive teacher-student relationships create an environment conducive to learning, where students feel safe to take risks, express themselves, and engage in their academic tasks.

Applying the principles of positive psychology to L2 instruction and educational research, it became evident that psychological support and caring interactions fostered feelings of closeness and engagement for both teachers and students, leading to overall well-being. Notably, when language instructors demonstrated care by establishing rapport, showing humor, displaying interpersonal affection, being accessible, and providing appropriate feedback, students experienced heightened feelings of empowerment, psychological resilience, and enthusiasm for their educational tasks (Derakhshan et al., 2022; Miller and Gkonou, 2023). This aligns with the broaden-and-build theory of positive emotions (Fredrickson, 2001), where positive teacher-student relationships expand students’ “momentary thought-action repertoires,” increasing their dedication to academic commitments through feelings of happiness, affection, and excitement. In addition, in the L2 setting, students’ well-being is enhanced as they become more engaged in pursuing their educational goals when instructors create an enjoyable learning atmosphere, attend to students’ needs, and foster intimate and enjoyable interactions with learners (Gkonou and Miller, 2019).

The second objective of this study was to examine the mediating role of grit in the relationship between perceived teacher caring and student wellbeing. SEM analysis approved the role of L2 grit as a significant mediator. The theoretical framework of grit suggests that individuals with higher levels of grit are better equipped to overcome obstacles, maintain focus, and sustain effort even in the face of challenges (Duckworth et al., 2007; Sudina et al., 2021). These individuals demonstrate resilience and a strong commitment to achieving their goals, which can have a profound impact on their overall wellbeing. When students feel supported by their teachers, they are encouraged to persist in their academic endeavors, even when faced with difficulties. The nurturing environment created by caring teachers promotes the development of qualities associated with grit, such as resilience, determination, and a growth mindset. As found by Banse and Palacios (2018), learners who perceived a high degree of care and class management from their instructors demonstrated greater grit. This girt, in turn affects students’ wellbeing, the finding which is consistent with previous research emphasizing the significance of grit in enhancing students’ wellbeing and classroom enjoyment (Elahi Shirvan et al., 2021; Resnik et al., 2021). Drawing inspiration from Fredrickson’s (2001) Broad-and-Build Theory, which posits that positive emotions broaden an individual’s mindset and foster enduring personal resources, it is anticipated that an instructor’s welcoming personality can create a nurturing and secure atmosphere for students, leading to higher levels of grit, satisfaction, and well-being throughout their educational journey.

## Conclusion

This study examined the impact of perceived teacher caring on student wellbeing in Chinese EFL students, with a focus on the mediating role of grit. The findings demonstrated a significant positive association between perceived teacher caring and student wellbeing, highlighting the crucial role of teacher-student relationships in fostering positive emotional experiences and overall wellbeing among EFL students in the Chinese context. Furthermore, grit was found to mediate the relationship between teacher caring and student wellbeing, suggesting that students with higher levels of grit are more likely to experience greater wellbeing. These findings emphasize the significance of nurturing positive and supportive teacher-student relationships and fostering grit among EFL students to enhance their overall wellbeing.

The findings of this study carry significant implications for educators, policymakers, and researchers in the field of EFL education. First and foremost, the results emphasize the pivotal role of teachers in fostering student wellbeing. It is crucial for educators to prioritize the development of positive and supportive teacher-student relationships as a foundational aspect of effective teaching. When teachers exhibit care and support for their students, they create a nurturing and inclusive classroom environment that directly contributes to student wellbeing.

Secondly, the mediating role of grit suggests that educators should also focus on cultivating grit among EFL students. Grit, characterized by perseverance and unwavering passion for long-term goals, enhances student resilience and determination, enabling them to flourish academically while experiencing greater overall wellbeing. By integrating strategies and interventions that promote grit, such as goal-setting, self-reflection, and fostering a growth mindset, educators can empower students to overcome challenges and persist in their academic pursuits. Furthermore, the study underscores the need for educational institutions to provide adequate support and resources for both teachers and students. Implementing professional development programs can equip teachers with the necessary skills and strategies to enhance their caring behaviors and foster positive relationships with students. Additionally, schools should offer resources and interventions that promote students’ grit development, such as mentoring programs, academic support, and character education initiatives.

Lastly, this research contributes to the existing knowledge base on teacher-student relationships, wellbeing, and grit in the EFL context. It enriches our understanding of the unique dynamics and factors that influence student wellbeing among Chinese EFL students. To build upon these findings, future research should continue to explore additional factors that may impact student wellbeing and further investigate the mechanisms through which teacher-student relationships and grit contribute to overall student wellbeing. By deepening our understanding in these areas, educators and policymakers can better tailor interventions and initiatives to promote students’ holistic development and academic success in the EFL setting.

However, it is essential to acknowledge several limitations that can provide context to the findings. Firstly, the study’s scope is specific to Chinese EFL students, and caution should be exercised when applying the results to students from diverse cultural backgrounds or educational settings. It is crucial to recognize that different contexts may influence the dynamics of teacher-student relationships and student wellbeing. Secondly, the study’s cross-sectional design has limitations in establishing causal relationships. For a more comprehensive understanding of the mediating role of grit and the impact of teacher caring on student wellbeing over time, longitudinal or experimental designs would provide more robust evidence. Additionally, the data collection relied on self-report questionnaires, making it vulnerable to response biases like social desirability and recall biases. Although self-report measures provide valuable insights, future research could incorporate multiple data sources and objective measures to ensure a more accurate and well-rounded evaluation. Moreover, it is important to note that while the scales used in this study were selected based on their relevance to the research objectives, not all of them were directly validated in the Chinese language or specific to the EFL context. Although efforts were made to ensure their items’ intelligibility for the participants, it should be acknowledged that these scales may not fully capture the nuances of the Chinese EFL educational environment.

Notably, the comprehensive validation of these adapted instruments across diverse cultures and languages presents a complex undertaking that exceeds the scope of this research. While the pre-testing phase provided valuable insights into the suitability and clarity of the instruments within the specific context of our study, the rigorous establishment of psychometric properties—such as validity and reliability—across various cultural and linguistic backgrounds necessitates an intricate process. This process entails larger sample sizes, expert reviews, and extensive psychometric analyses.

The primary focus of the present study was to investigate the mediation model involving teacher caring, grit, and student wellbeing within the Chinese EFL student population. The insights gleaned from this study significantly contribute to our understanding of these dynamics within this specific context. However, it is essential to approach the interpretation of results with the awareness that the full establishment of psychometric robustness for the adapted instruments has limitations. Future research endeavors hold promise in addressing these limitations by embarking on comprehensive cross-cultural validation studies. Such studies would delve deeply into the psychometric properties of the adapted instruments, thereby enhancing the generalizability of our findings to diverse populations.

Finally, although the study explored the mediating role of grit, it is essential to acknowledge that mediation does not imply causation. There may be other unmeasured variables or alternative mediation models that contribute to the observed relationships. Further investigation into the underlying mechanisms and alternative explanations is warranted.

## Data availability statement

The raw data supporting the conclusions of this article will be made available by the authors, without undue reservation. Requests to access these datasets should be directed to GZ, 2211026@stu.zykj.edu.cn.

## Ethics statement

The studies involving humans were approved by College of education, Zhongyuan Institute of Science and Technology, Zhengzhou, China. The studies were conducted in accordance with the local legislation and institutional requirements. The participants provided their written informed consent to participate in this study.

## Author contributions

GZ: Conceptualization, Data curation, Formal analysis, Investigation, Methodology, Project administration, Resources, Software, Validation, Visualization, Writing – original draft, Writing – review & editing, Project administration.

## Funding

The author declares that no financial support was received for the research, authorship, and/or publication of this article.

## Conflict of interest

The author declares that the research was conducted in the absence of any commercial or financial relationships that could be construed as a potential conflict of interest.

## Publisher’s note

All claims expressed in this article are solely those of the authors and do not necessarily represent those of their affiliated organizations, or those of the publisher, the editors and the reviewers. Any product that may be evaluated in this article, or claim that may be made by its manufacturer, is not guaranteed or endorsed by the publisher.

## References

[ref1] Akos P. Kretchmar J. (2017). Investigating grit at a non-cognitive predictor of college success. Rev. High. Educ. 40, 163–186. doi: 10.1353/rhe.2017.0000

[ref2] Alamer A. (2021). Grit and language learning: construct validation of L2-Grit scale and its relation to later vocabulary knowledge. Educ. Psychol. 41, 544–562. doi: 10.1080/01443410.2020.1867076

[ref3] Alamer A. (2022). Having a single language interest autonomously predicts L2 achievement: Addressing the predictive validity of L2 grit. System 108:102850. doi: 10.1016/j.system.2022.102850

[ref4] Anderson D. L. Graham A. P. (2016). Improving student wellbeing: Having a say at school. Sch. Eff. Sch. Improv. 27, 348–366. doi: 10.1080/09243453.2015.1084336

[ref5] Banse H. Palacios N. (2018). Supportive classrooms for Latino English language learners: Grit, ELL status, and the classroom context. J. Educ. Res. 111, 645–656. doi: 10.1080/00220671.2017.1389682

[ref6] Braun S. S. Schonert-Reichl K. A. Roeser R. W. (2020). Effects of teachers' emotion regulation, burnout, and life satisfaction on student well-being. J. Appl. Dev. Psychol. 69:101151. doi: 10.1016/j.appdev.2020.101151

[ref7] Bücker S. Nuraydin S. Simonsmeier B. A. Schneider M. Luhmann M. (2018). Subjective well-being and academic achievement: a meta-analysis. J. Res. Pers. 74, 83–94. doi: 10.1016/j.jrp.2018.02.007, PMID: 30473200

[ref8] Cakir S. G. (2015). The effects of teacher immediacy and student burnout on empowerment and resistance among Turkish pre-service teachers. Learn. Individ. Differ. 40, 170–175. doi: 10.1016/j.lindif.2015.05.002

[ref9] Changlek A. Palanukulwong T. (2015). Motivation and grit: Predictors of language learning achievement. Veridian E-Journal 8, 23–36.

[ref10] Cheng X. Liu Y. (2022). Student engagement with teacher written feedback: Insights from low-proficiency and high-proficiency L2 learners. System 109:102880. doi: 10.1016/j.system.2022.102880

[ref11] Clark K. N. Malecki C. K. (2019). Academic Grit Scale: Psychometric properties and associations with achievement and life satisfaction. J. Sch. Psychol. 72, 49–66. doi: 10.1016/j.jsp.2018.12.001, PMID: 30819462

[ref12] Cohen S. Wills T. A. (1985). Stress, social support, and the buffering hypothesis. Psychol. Bull. 98:310. doi: 10.1037/0033-2909.98.2.310, PMID: 3901065

[ref13] Collie R. J. (2022). Instructional support, perceived social-emotional competence, and students’ behavioral and emotional well-being outcomes. Educ. Psychol. 42, 4–22. doi: 10.1080/01443410.2021.1994127, PMID: 20350027

[ref14] Credé M. (2018). What shall we do about grit? A critical review of what we know and what we don’t know. Educ. Res. 47, 606–611. doi: 10.3102/0013189X18801322

[ref15] Credé M. Tynan M. C. (2021). Should language acquisition researchers study “grit”? A cautionary note and some suggestions. J. Psychol. Lang. Learn. 3, 37–44. doi: 10.52598/jpll/3/2/3

[ref16] Credé M. Tynan M. C. Harms P. D. (2017). Much ado about grit: a meta-analytic synthesis of the grit literature. J. Pers. Soc. Psychol. 113:492. doi: 10.1037/pspp0000102, PMID: 27845531

[ref17] Derakhshan A. Doliński D. Zhaleh K. Enayat M. J. Fathi J. (2022). A mixed-methods cross-cultural study of teacher care and teacher-student rapport in Iranian and Polish University students’ engagement in pursuing academic goals in an L2 context. System 106:102790. doi: 10.1016/j.system.2022.102790

[ref18] Dewaele J. M. Chen X. Padilla A. M. Lake J. (2019). The flowering of positive psychology in foreign language teaching and acquisition research. Front. Psychol. 10:2128. doi: 10.3389/fpsyg.2019.02128 31607981 PMC6769100

[ref19] Dewaele J.-M. Gkonou C. Mercer S. (2018). Do ESL/EFL teachers’ emotional intelligence, teaching experience, proficiency and gender, affect their classroom practice?. Emotions in second language teaching. ed. Dios Martínez Agudo J. de. Berlin: Springer, 125–141.

[ref20] Diener E. D. Emmons R. A. Larsen R. J. Griffin S. (1985). The satisfaction with life scale. J. Pers. Assess. 49, 71–75. doi: 10.1207/s15327752jpa4901_13 16367493

[ref21] Duckworth A. L. Peterson C. Matthews M. D. Kelly D. R. (2007). Grit: perseverance and passion for long-term goals. J. Pers. Soc. Psychol. 92:1087. doi: 10.1037/0022-3514.92.6.1087, PMID: 17547490

[ref22] Duckworth A. L. Quinn P. D. (2009). Development and validation of the Short Grit Scale (GRIT–S). J. Pers. Assess. 91, 166–174. doi: 10.1080/00223890802634290, PMID: 19205937

[ref23] Duckworth A. L. Quinn P. D. Tsukayama E. (2021). Revisiting the factor structure of grit: a commentary on Duckworth and Quinn (2009). J. Pers. Assess. 103, 573–575. doi: 10.1080/00223891.2021.1942022, PMID: 34254861

[ref24] Elahi Shirvan M. Taherian T. Shahnama M. Yazdanmehr E. (2021). A longitudinal study of foreign language enjoyment and L2 grit: A latent growth curve modeling. Front. Psychol. 12:720326. doi: 10.3389/fpsyg.2021.720326 34526939 PMC8435725

[ref25] Fabris M. A. Roorda D. Longobardi C. (2022). Student-teacher relationship quality research: Past, present and future. Front. Educ. 7:1049115. doi: 10.3389/feduc.2022.1049115

[ref26] Fathi J. Pawlak M. Kruk M. Naderi M. (2023a). Modelling boredom in the EFL context: an investigation of the role of coping self-efficacy, mindfulness, and foreign language enjoyment. Lang. Teach. Res. doi: 10.1177/13621688231182176 (in press).

[ref27] Fathi J. Pawlak M. Mehraein S. Hosseini H. M. Derakhshesh A. (2023b). Foreign language enjoyment, ideal L2 self, and intercultural communicative competence as predictors of willingness to communicate among EFL learners. System 115:103067. doi: 10.1016/j.system.2023.103067

[ref28] Fredrickson B. L. (2001). The role of positive emotions in positive psychology: The broaden-and-build theory of positive emotions. Am. Psychol. 56:218. doi: 10.1037/0003-066X.56.3.218, PMID: 11315248 PMC3122271

[ref29] Gabryś-Barker D. (2016). “Caring and sharing in the foreign language class: on a positive classroom climate” in Positive psychology perspectives on foreign language learning and teaching. eds. Gabryś-Barker D. Gałajda D. (New York: Springer), 155–174.

[ref30] Gallagher E. K. Dever B. V. Hochbein C. DuPaul G. J. (2019). Teacher caring as a protective factor: The effects of behavioral/emotional risk and teacher caring on office disciplinary referrals in middle school. Sch. Ment. Heal. 11, 754–765. doi: 10.1007/s12310-019-09318-0

[ref31] Gkonou C. Miller E. R. (2019). Caring and emotional labour: Language teachers’ engagement with anxious learners in private language school classrooms. Lang. Teach. Res. 23, 372–387. doi: 10.1177/1362168817728739

[ref34] Greenier V. Derakhshan A. Fathi J. (2021). Emotion regulation and psychological well-being in teacher work engagement: a case of British and Iranian English language teachers. System 97:102446. doi: 10.1016/j.system.2020.102446

[ref35] Harding S. Morris R. Gunnell D. Ford T. Hollingworth W. Tilling K.. (2019). Is teachers’ mental health and wellbeing associated with students’ mental health and wellbeing? J. Affect. Disord. 242, 180–187. doi: 10.1016/j.jad.2018.08.080, PMID: 30189355

[ref36] Hayes A. F. (2009). Beyond Baron and Kenny: Statistical mediation analysis in the new millennium. Commun. Monogr. 76, 408–420. doi: 10.1080/03637750903310360

[ref37] Hu L. T. Bentler P. M. (1999). Cutoff criteria for fit indexes in covariance structure analysis: conventional criteria versus new alternatives. Struct. Equ. Model. Multidiscip. J. 6, 1–55. doi: 10.1080/10705519909540118, PMID: 36787513

[ref38] Khajavy G. H. Aghaee E. (2022). The contribution of grit, emotions and personal bests to foreign language learning. J. Multiling. Multicult. Dev. 1–15. doi: 10.1080/01434632.2022.2047192 (in press).

[ref39] Khajavy G. H. MacIntyre P. D. Hariri J. (2021). A closer look at grit and language mindset as predictors of foreign language achievement. Stud. Second. Lang. Acquis. 43, 379–402. doi: 10.1017/S0272263120000480

[ref40] Kline R. B. (2023). Principles and practice of structural equation modeling. New York: Guilford publications.

[ref41] Kramer B. McLean S. Shepherd Martin E. S. (2018). Student grittiness: a pilot study investigating scholarly persistence in EFL classrooms. Osaka: Osaka Jogakuin College.

[ref42] Lake J. (2013). Positive L2 self: linking positive psychology with L2 motivation. Language learning motivation in Japan. eds. Apple M. Silva D. Da Fellner T. Bristol: Multilingual Matters, 225–244.

[ref43] Laletas S. Reupert A. (2016). Exploring pre-service secondary teachers’ understanding of care. Teach. Teach. 22, 485–503. doi: 10.1080/13540602.2015.1082730

[ref44] Lavy S. Naama-Ghanayim E. (2020). Why care about caring? Linking teachers’ caring and sense of meaning at work with students’ self-esteem, well-being, and school engagement. Teach. Teach. Educ. 91:103046. doi: 10.1016/j.tate.2020.103046

[ref45] Lewis J. L. Ream R. K. Bocian K. M. Cardullo R. A. Hammond K. A. Fast L. A. (2012). Con cariño: Teacher caring, math self-efficacy, and math achievement among Hispanic English learners. Teach. Coll. Rec. 114, 1–42. doi: 10.1177/016146811211400701

[ref46] Li C. (2020). A positive psychology perspective on Chinese EFL students’ trait emotional intelligence, foreign language enjoyment and EFL learning achievement. J. Multiling. Multicult. Dev. 41, 246–263. doi: 10.1080/01434632.2019.1614187

[ref47] Li C. Dewaele J. M. (2021). How classroom environment and general grit predict foreign language classroom anxiety of Chinese EFL students. J. Psychol. Lang. Learn. 3, 86–98. doi: 10.52598/jpll/3/2/6

[ref48] Lin S. Fabris M. A. Longobardi C. (2022). Closeness in student–teacher relationships and students’ psychological well-being: the mediating role of hope. J. Emot. Behav. Disord. 30, 44–53. doi: 10.1177/10634266211013756

[ref49] Little T. D. Rhemtulla M. (2013). Planned missing data designs for developmental researchers. Child Dev. Perspect. 7, 199–204. doi: 10.1111/cdep.12043, PMID: 36321557

[ref50] Longobardi C. Settanni M. Lin S. Fabris M. A. (2021). Student–teacher relationship quality and prosocial behaviour: the mediating role of academic achievement and a positive attitude towards school. Br. J. Educ. Psychol. 91, 547–562. doi: 10.1111/bjep.12378, PMID: 32920835

[ref51] MacIntyre P. D. Gregersen T. Mercer S. (Eds). (2016). “Conclusion,” in Positive psychology in SLA. Bristol: Multilingual Matters, 374–379.

[ref52] MacIntyre P. D. Gregersen T. Mercer S. (2019). Setting an agenda for positive psychology in SLA: theory, practice, and research. Mod. Lang. J. 103, 262–274. doi: 10.1111/modl.12544

[ref53] McCain B. (2017). Effects of teacher grit on student grit and reading achievement: a mixed-methods study. Indiana, PA: Indiana University of Pennsylvania.

[ref54] Mercer S. Dörnyei Z. (2020). Engaging language learners in contemporary classrooms. Cambridge: Cambridge University Press.

[ref55] Mercer S. Gkonou C. (2020). “Relationships and good language teachers” in Lessons from good language teachers. eds. Griffiths C. Tajeddin Z. (Cambridge: Cambridge University Press), 164–174.

[ref56] Mercer S. Gregersen T. (2020). Teacher wellbeing. Oxford: Oxford University Press.

[ref57] Mercer S. MacIntyre P. Gregersen T. Talbot K. (2018). Positive language education: Combining positive education and language education. Theory Pract. Second Lang. Acquis. 4, 11–31.

[ref58] Mikami H. (2023). Revalidation of the L2-Grit scale: A conceptual replication of Teimouri, Y., Plonsky, L., & Tabandeh, F. (2022). L2 grit: Passion and perseverance for second-language learning. Lang. Teach., 1–16. doi: 10.1017/S0261444822000544

[ref59] Miller E. R. Gkonou C. (2023). Exploring teacher caring as a “happy object” in language teacher accounts of happiness. Appl. Linguis. 44, 328–346. doi: 10.1093/applin/amac034

[ref60] Mohammad Hosseini H. Fathi J. Derakhshesh A. Mehraein S. (2022). A model of classroom social climate, foreign language enjoyment, and student engagement among English as a foreign language learners. Front. Psychol. 13:933842. doi: 10.3389/fpsyg.2022.933842, PMID: 36059776 PMC9428561

[ref61] Morinaj J. Hascher T. (2019). School alienation and student well-being: a cross-lagged longitudinal analysis. Eur. J. Psychol. Educ. 34, 273–294. doi: 10.1007/s10212-018-0381-1

[ref62] Moskowitz G. Caring and sharing in the foreign language class: a sourcebook on humanistic technique (No. 407 M911c). Boston, MA: Heinle. (1978).

[ref63] Muller C. (2001). The role of caring in the teacher-student relationship for at-risk students. Sociol. Inq. 71, 241–255. doi: 10.1111/j.1475-682X.2001.tb01110.x

[ref64] Mystkowska-Wiertelak A. (2022). Teachers’ accounts of learners’ engagement and disaffection in the language classroom. Lang. Learn. J. 50, 393–405. doi: 10.1080/09571736.2020.1800067

[ref65] Nalipay M. J. N. King R. B. Mordeno I. G. Wang H. (2022). Are good teachers born or made? Teachers who hold a growth mindset about their teaching ability have better well-being. Educ. Psychol. 42, 23–41. doi: 10.1080/01443410.2021.2001791, PMID: 37516820

[ref66] Noddings N. (1984). Caring: a feminine approach to ethics and moral education. Berkeley, CA: University of California Press

[ref67] Noddings N. (2012). The caring relation in teaching. Oxf. Rev. Educ. 38, 771–781. doi: 10.1080/03054985.2012.745047, PMID: 37610998

[ref68] Oxford R. Khajavy G. H. (2021). Exploring Grit: " Grit Linguistics" and research on domain-general grit and L2 grit. J. Psychol. Lang. Learn. 3, 7–36. doi: 10.52598/jpll/3/2/2, PMID: 36405217

[ref69] Pianta R. C. Hamre B. K. Allen J. P. (2012). “Teacher-student relationships and engagement: Conceptualizing, measuring, and improving the capacity of classroom interactions” in Handbook of research on student engagement (Boston, MA: Springer US), 365–386.

[ref70] Pishghadam R. Derakhshan A. Zhaleh K. (2019). The interplay of teacher success, credibility, and stroke with respect to EFL students’ willingness to attend classes. Pol. Psychol. Bull. 50, 284–292. doi: 10.24425/ppb.2019.131001

[ref71] Pishghadam R. Naji Meidani E. Khajavy G. H. (2015). Language teachers' conceptions of intelligence and their roles in teacher care and teacher feedback. Austral. J. Teach. Educ. 40, 60–82. doi: 10.14221/ajte.2015v40n1.4

[ref72] Postigo Á. Cuesta M. García-Cueto E. Menéndez-Aller Á. González-Nuevo C. Muñiz J. (2021). Grit assessment: is one dimension enough? J. Pers. Assess. 103, 786–796. doi: 10.1080/00223891.2020.1848853, PMID: 33236925

[ref73] Ramberg J. Låftman S. B. Almquist Y. B. Modin B. (2019). School effectiveness and students’ perceptions of teacher caring: a multilevel study. Improv. Sch. 22, 55–71. doi: 10.1177/1365480218764693

[ref74] Resnik P. Dewaele J. M. (2020). Trait emotional intelligence, positive and negative emotions in first and foreign language classes: A mixed-methods approach. System 94:102324. doi: 10.1016/j.system.2020.102324

[ref75] Resnik P. Moskowitz S. Panicacci A. (2021). Language learning in crisis mode: the connection between LX grit, trait emotional intelligence and learner emotions. J. Psychol. Lang. Learn. 3, 99–117. doi: 10.52598/jpll/3/2/7

[ref76] Robins S. (2019). Academic achievement and retention among ESL learners: a study of grit in an online context (Doctoral dissertation). Carrollton, GA: University of West Georgia.

[ref77] Ryff C. D. (1989). Happiness is everything, or is it? Explorations on the meaning of psychological well-being. J. Pers. Soc. Psychol. 57:1069. doi: 10.1037/0022-3514.57.6.1069, PMID: 37604657

[ref78] Seligman M. E. (2011). Flourish: a visionary new understanding of happiness and well-being. New York, NY: Simon and Schuster.

[ref79] Sudina E. Plonsky L. (2021). Academic perseverance in foreign language learning: an investigation of language-specific grit and its conceptual correlates. Mod. Lang. J. 105, 829–857. doi: 10.1111/modl.12738

[ref80] Sudina E. Vernon T. Foster H. Del Villano H. Hernandez S. Beck D.. (2021). Development and initial validation of the L2-teacher grit scale. TESOL Q. 55, 156–184. doi: 10.1002/tesq.581

[ref81] Sulla F. Aquino A. Rollo D. (2022). University students' online learning during COVID-19: the role of grit in academic performance. Front. Psychol. 13:825047. doi: 10.3389/fpsyg.2022.825047, PMID: 35222206 PMC8866721

[ref82] Sulla F. Renati R. Bonfiglio S. Rollo D. (2018). “Italian students and the Grit-S: a self-report questionnaire for measuring perseverance and passion for long-term goals” in 2018 IEEE International Symposium on Medical Measurements and Applications (MeMeA) (New York: IEEE), 1–5.

[ref83] Sulla F. Rollo D. (2023). The effect of a short course on a group of Italian primary school teachers’ rates of praise and their pupils’ on-task behaviour. Educ. Sci. 13:78. doi: 10.3390/educsci13010078

[ref84] Sun Y. (2021). The effect of teacher caring behavior and teacher praise on students’ engagement in EFL classrooms. Front. Psychol. 12:3840. doi: 10.3389/fpsyg.2021.746871 PMC8478015 34594287

[ref85] Tabachnick B. G. Fidell L. S. Ullman J. B. (2013). Using multivariate statistics, 6, 497–516. Boston, MA: Pearson.

[ref86] Talbot K. Mercer S. (2018). Exploring university ESL/EFL teachers’ emotional well-being and emotional regulation in the United States, Japan and Austria. Chin. J. Appl. Linguist. 41, 410–432. doi: 10.1515/cjal-2018-0031

[ref87] Teimouri Y. Plonsky L. Tabandeh F. (2022). L2 grit: Passion and perseverance for second-language learning. Lang. Teach. Res. 26, 893–918. doi: 10.1177/1362168820921895

[ref88] Teven J. J. (2007). Teacher caring and classroom behavior: Relationships with student affect and perceptions of teacher competence and trustworthiness. Commun. Q. 55, 433–450. doi: 10.1080/01463370701658077

[ref89] Teven J. J. McCroskey J. C. (1997). The relationship of perceived teacher caring with student learning and teacher evaluation. Commun. Educ. 46, 1–9. doi: 10.1080/03634529709379069, PMID: 34294263

[ref90] Upsher R. Nobili A. Hughes G. Byrom N. (2022). A systematic review of interventions embedded in curriculum to improve university student wellbeing. Educ. Res. Rev. 37:100464. doi: 10.1016/j.edurev.2022.100464, PMID: 37131375

[ref91] Wang Y. Derakhshan A. Zhang L. J. (2021). Researching and practicing positive psychology in second/foreign language learning and teaching: the past, current status and future directions. Front. Psychol. 12:731721. doi: 10.3389/fpsyg.2021.731721, PMID: 34489835 PMC8417049

[ref92] Wei H. Gao K. Wang W. (2019). Understanding the relationship between grit and foreign language performance among middle school students: the roles of foreign language enjoyment and classroom environment. Front. Psychol. 10:1508. doi: 10.3389/fpsyg.2019.01508 31333541 PMC6624730

[ref93] Wei R. Liu H. Wang S. (2020). Exploring L2 grit in the Chinese EFL context. System 93:102295. doi: 10.1016/j.system.2020.102295

[ref94] Xie F. Derakhshan A. (2021). A conceptual review of positive teacher interpersonal communication behaviors in the instructional context. Front. Psychol. 12:708490. doi: 10.3389/fpsyg.2021.708490, PMID: 34335424 PMC8319622

[ref95] Yan P. (2021). Chinese EFL students' perceptions of classroom justice: The impact of teachers' caring and immediacy. Front. Psychol. 12:767008. doi: 10.3389/fpsyg.2021.767008, PMID: 34744945 PMC8566534

[ref96] Yang S. Azari Noughabi M. Jahedizadeh S. (2022). Modelling the contribution of English language learners’ academic buoyancy and self-efficacy to L2 grit: evidence from Iran and China. J. Multiling. Multicult. Dev. 1–17. doi: 10.1080/01434632.2022.2062368 (in press).

[ref97] Zhang L. J. Fathi J. Mohammaddokht F. (2023). Predicting teaching enjoyment from teachers’ perceived school climate, self-efficacy, and psychological wellbeing at work: EFL teachers. Percept. Mot. Skills. doi: 10.1177/00315125231182269 (in press). PMC10552353 37395156

[ref98] Zhang L. J. Saeedian A. Fathi J. (2022). Testing a model of growth mindset, ideal L2 self, boredom, and WTC in an EFL context. J. Multiling. Multicult. Dev. 1–16. doi: 10.1080/01434632.2022.2062366 (in press).

